# A proposal for a new classification of coracobrachialis muscle morphology

**DOI:** 10.1007/s00276-021-02700-1

**Published:** 2021-02-09

**Authors:** Bartłomiej Szewczyk, Michał Polguj, Friedrich Paulsen, Michał Podgórski, Fabrice Duparc, Piotr Karauda, Łukasz Olewnik

**Affiliations:** 1 Department of Anatomical Dissection and Donation, Medical University of Lodz, Lodz, Poland; 2 Department of Normal and Clinical Anatomy, Chair of Anatomy and Histology, Medical University of Lodz, Lodz, Poland; 3 Polish Mother’s Memorial Hospital Research Institute, Lodz, Poland; 4 Institute of Functional and Clinical Anatomy, Erlangen, Germany; 5 Department of Topographic Anatomy and Operative Surgery, Sechenov University, Moscow, Russia; 6 Laboratory of Anatomy, Faculty of Medicine, Rouen University, Mont-Saint-Aignan, France

**Keywords:** Coracobrachialis muscle, Coracobrachialis muscle, Musculocutaneous nerve, Median nerve, New classification

## Abstract

**Introduction:**

The coracobrachialis muscle (CRM) originates from the apex of the coracoid process, in common with the short head of the biceps brachii muscle, and from the intermuscular septum. It inserts to the medial part of the humerus between the attachment of the medial head of the triceps brachii and the brachial muscle. Both the proximal and distal attachments of the CRM, as well as its relationship with the musculocutaneus nerve, demonstrate morphological variability.

**Material and methods:**

One hundred and one upper limbs (52 left, and 49 right) fixed in 10% formalin solution were examined.

**Results:**

Three main types, with subtypes, were identified. The most common was Type I (49.5), characterized by a single muscle belly with a classical origin from the coracoid process, medially and posteriorly to the tendon of the biceps brachii. Type II (42.6%), characterized by two heads, was divided into two subtypes (A-B) depending on its origin: Type IIA, where one head originated from the coracoid process posteriorly to the tendon of the biceps brachii and the second head from the short head of the biceps brachii, and Type IIB, in which both heads originated from the coracoid process; however, the superficial head fused with the insertion of a short head of the biceps brachii, while the deep head was directly originating. Finally, Type III (7.9%) was characterized by three heads: two originated from the coracoid process (superficial and deep), and the third from a short head of the biceps brachii. Two types of insertion and two types of musculocutaneous nerve (MCN) relative to CRM could be distinguished.

**Conclusion:**

An adapted classification is needed for all clinicians working in this area, as well as for anatomists. The CRM demonstrates morphological variability in both its proximal and distal attachments, as well as the variable course of the MCN relative to the CRM.

**What is known about this subject "and" What this study adds to existing knowledge:**

Not much is known about the variability of coracobrachialis muscle. The present paper introduces a completely new classification, both clinical and anatomical.

## Introduction

The coracoid process serves as an important anchor for several tendinous and ligamentous structures. These include, medially to laterally, the tendons of the pectoralis minor, coracobrachialis (CRM), and the short head of the biceps brachii muscles (shBB), and, laterally to medially, the coracohumeral, coracoacromial, coracoclavicular, and superior transverse scapular ligaments. The CRM and shBB share a common origin on the apex of the coracoid process of the scapula. The CRM inserts into the medial surface of the humerus, between the attachments of the triceps brachii and brachialis muscles by means of a short, flat tendon [1]. The CRM serves to flex and adduct the arm at the glenohumeral joint, and to resist deviation of the arm from the frontal plane during abduction [1].

The brachial plexus and the major axillary vessels run medially and inferiorly to the coracoid process; they begin to divide into branches from the medial, lateral, and posterior cords at the level of the coracoid process, anterior to the inferior glenoid [1]. The lateral cord of the brachial plexus gives rise to the musculocutaneous nerve (MCN), which contains fibers from the C5–C7 ventral rami. The MCN passes through the CRM and descends between the biceps brachii and brachialis muscles, innervating both of them [1].

A number of authors have described variations in the CRM and MCN [2–8]. Most of these classifications are based on whether the MCN pierces CRM or not; however, that of Loukas et al. [7] includes the relationship between the MCN, the median nerve (MN) and the CRM. In contrast, Hayashi et al. [9] examined the relationship between the communicating branch and the transposed innervation of the brachial flexors to the median nerve. Only El-Naggar et al. [2] and Ilayperuma et al. [10], examined the morphology of the muscle. The remaining studies mainly concern reports of isolated cases, such as examples of accessory slips of the muscle inserting to the medial epicondyle and medial supracondylar ridge of the humerus, or additional heads or bellies [6, 11–14].

A good understanding of the anatomical relationship between the coracoid process and the CRM, shBB and pectoralis minor, as well as other glenohumeral joint supporting structures, is needed to correctly interpret shoulder magnetic resonance (MR) imaging of this area, and when planning proper surgical procedures in this area.

The aim of the present study was to characterize possible variations in the morphology of the proximal and distal attachments of the CRM and to draw conclusions from this with regard to an accurate classification of the area that can be useful for planning surgical procedures in the region. It also should assess the relationship between CRM type and MCN course.

## Materials and methods

One hundred and one upper limbs (52 left, and 49 right) fixed in 10% formalin solution were examined. The mean age “at death” of the cadavers was 77.1 years (48–95), and the group comprised equal numbers of female and male adults (Central European population). The cadavers were the property of the Department of Anatomical Dissection and Donation, Medical University of Lodz, Poland, following donation to the university anatomy program. Any upper limbs with evidence of surgical intervention in the dissected area were excluded. All dissection of the shoulder and arm area were performed in accordance with an pre-established protocol [15–19].

Dissection began with the removal of the skin and superficial fascia from the area of the shoulder and medial side of the arm. The next step included lateral, medial and posterior cords of the brachial plexus visualization, as well as accurate visualization of both biceps brachii, CRM and brachialis muscle. Following this, all structures were thoroughly cleaned.

Upon dissection, the following morphological features of the CRM were assessed:

- The type of origin of the CRM
- The type of insertion of the CRM
- The relationship between CRM and MCN
- Morphometric measurements of the CRM and MCN.

When dissecting the CRM:

- Special attention was paid when cleaning the shBB as it has numerous connections to the CRM, and provides an origin for the CRM.
- when assessing the course of the MCN, the deep head of the CRM was often invisible at first sight: the area was thoroughly cleaned.

An electronic digital caliper was used for all measurements (Mitutoyo Corporation, Kawasaki-shi, Kanagawa, Japan), and each measurement was performed twice with an accuracy of up to 0.1 mm. The Bioethics Committee of the Medical University of Lodz (resolution RNN/1337/20/KE) approved the study protocol. The cadavers belong to the Department of Anatomical Dissection and Donation of the Medical University of Lodz, Poland.

### Statistical analysis

Statistica 13 software (StatSoft Polska, Cracow, Poland) was used for the statistical analysis. The following tests were applied:

- The Chi-square test to compare nominal data—differences of muscle, insertion and innervation types between each other and between body sides and sexes.
- The Shapiro–Wilk test to assess normality of the morphological measurments distribution. As the data was not normally distributed nonparametric tests were used.
- The Mann–Whitney test to compare morphological measurements between body sides, sex, and types of insertion and innervation.
- The Kruskal–Wallis ANOVA by ranks with dedicated post hoc test to compare measurements between muscle types.

A p-value lower than 0.05 was considered significant, with Bonferroni’s correction for multiple testing. The results are presented as mean and standard deviation unless otherwise stated.

## Results

**Fig. 1:**
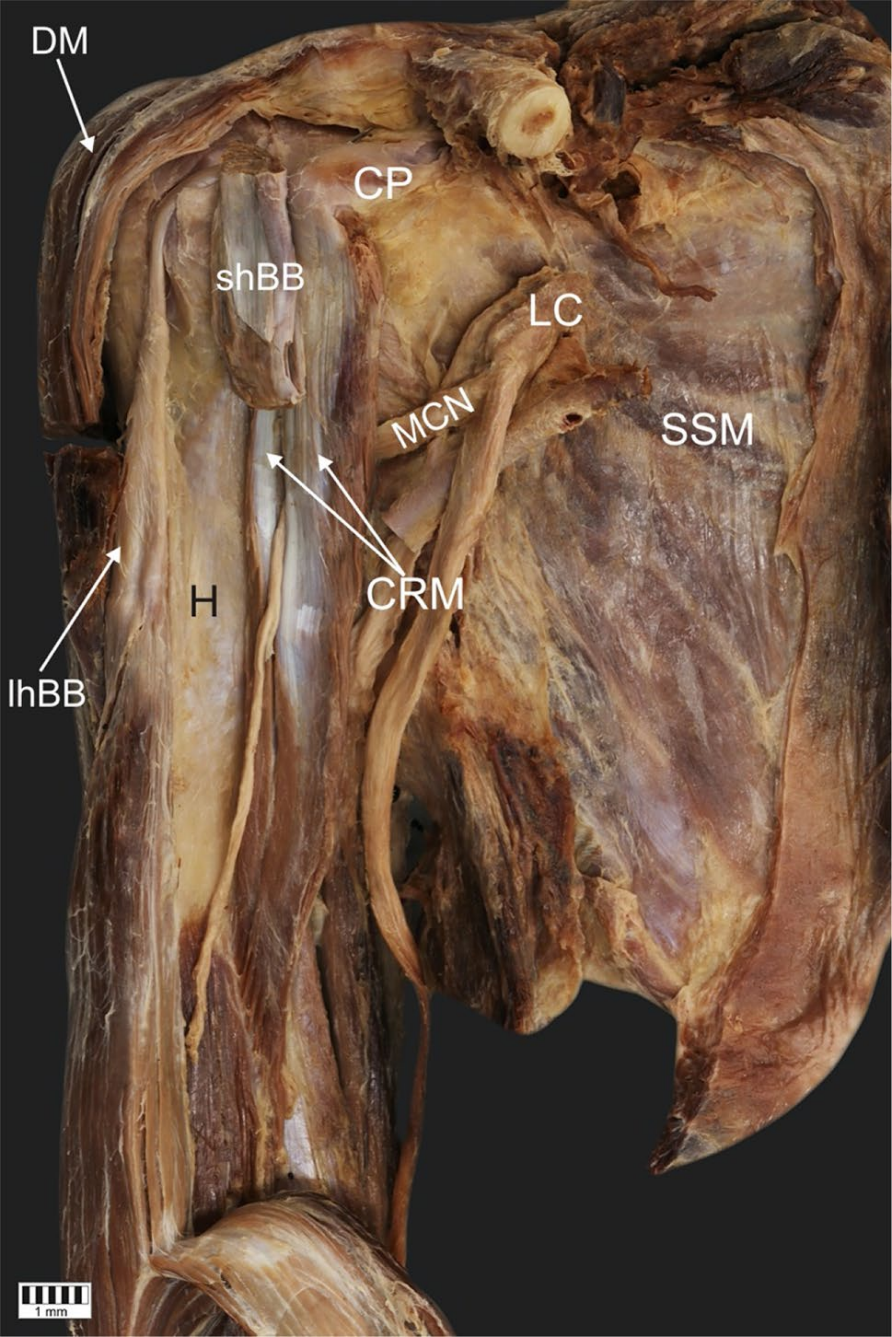
Type I of origin of the coracobrachialis muscle. Right arm. *DM* deltoid muscle, *CP* coracoid process of the scapula, *shBB* short head of the biceps brachii, *lhBB* long head of the biceps brachii, *LC* lateral cord of the brachial plexus, *H* humerus, *SSM* subscapularis muscle, *MCN* musculocutaneous nerve, *CRM* coracobrachialis muscle

**Fig. 2:**
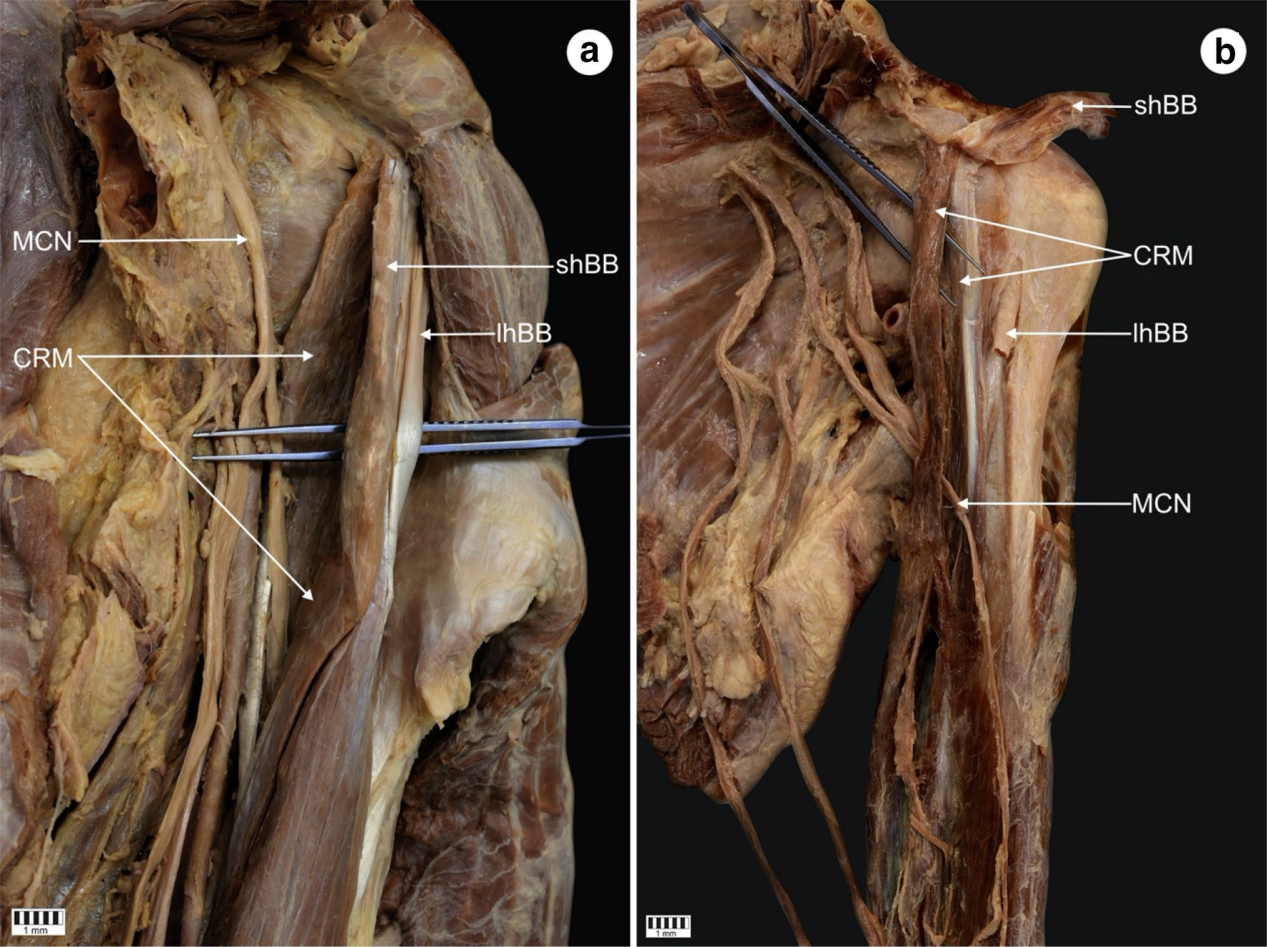
Type II of origin of the coracobrachialis muscle. Left arm. **a** Type IIa of the coracobrachialis muscle. *MCN* musculocutaneous nerve, *CRM* coracobrachialis muscle, *shBB* short head of the biceps brachii, *lhBB* long head of the biceps brachii, *CP* coracoid process. **b** Type IIb of origin of the coracobrachialis muscle. *CRM* coracobrachialis muscle, *shBB* short head of the biceps brachii, *lhBB* long head of the biceps brachii, *MCN* musculocutaneous nerve, *CP* coracoid process

**Fig. 3:**
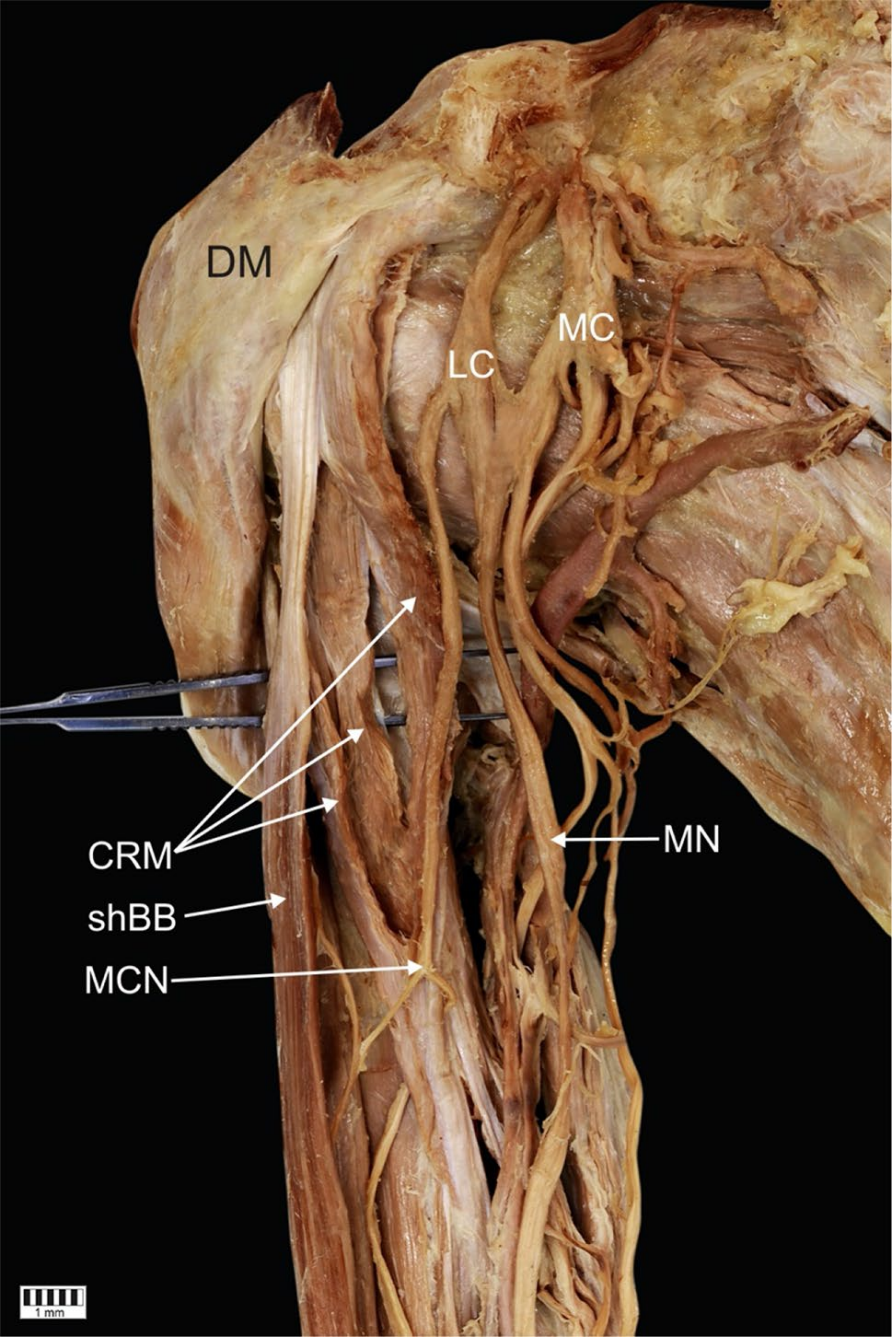
Type III of origin of the coracobrachialis muscle. Right arm. *DM* deltoid muscle, *LC* lateral cord of the brachial plexus, *MC* medial cord of the brachial plexus, *CRM* coracobrachialis muscle, *shBB* short head of the biceps brachii, *MCN* musculocutaneous nerve, *MN* median nerve, *CP* coracoid process

**Fig. 4:**
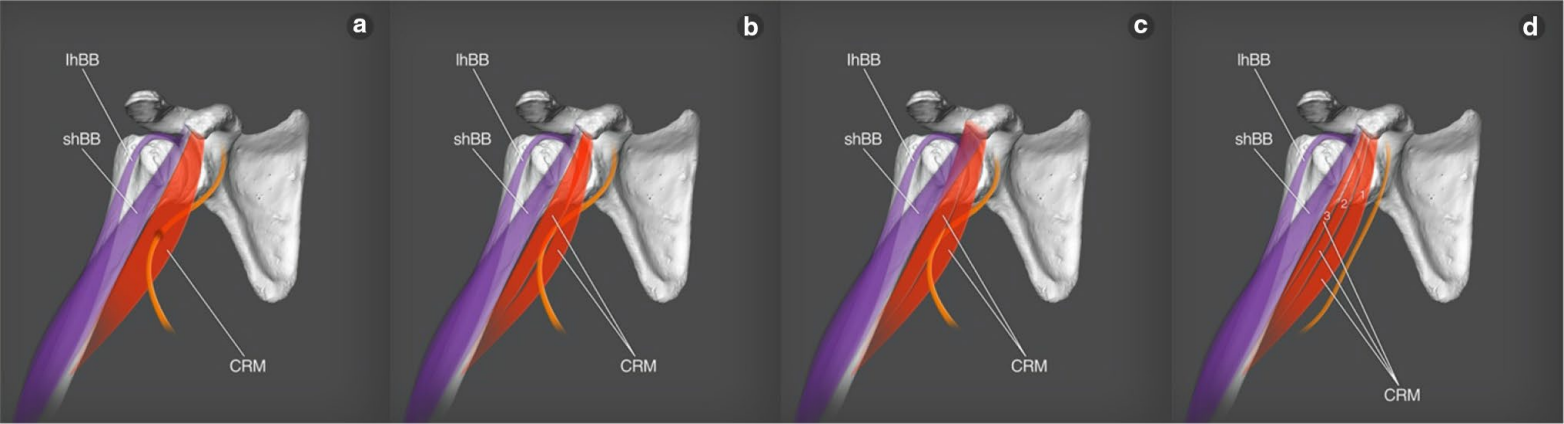
Scheme of types origin of coracobrachialis muscle. **a** scheme of Type I origin of coracobrachialis muscle *lhBB* long head of the biceps brachii *shBB* short head of the biceps brachii *CRM* coracobrachialis muscle **b** scheme of Type II a origin of coracobrachialis muscle *lhBB* long head of the biceps brachii *shBB* short head of the biceps brachii *CRM* coracobrachialis muscle **c** scheme of Type II b origin of coracobrachialis muscle *lhBB* long head of the biceps brachii *shBB* short head of the biceps brachii *CRM* coracobrachialis muscle **d** scheme of Type III origin of coracobrachialis muscle *lhBB* long head of the biceps brachii *shBB* short head of the biceps brachii *CRM* coracobrachialis muscle

**Fig. 5:**
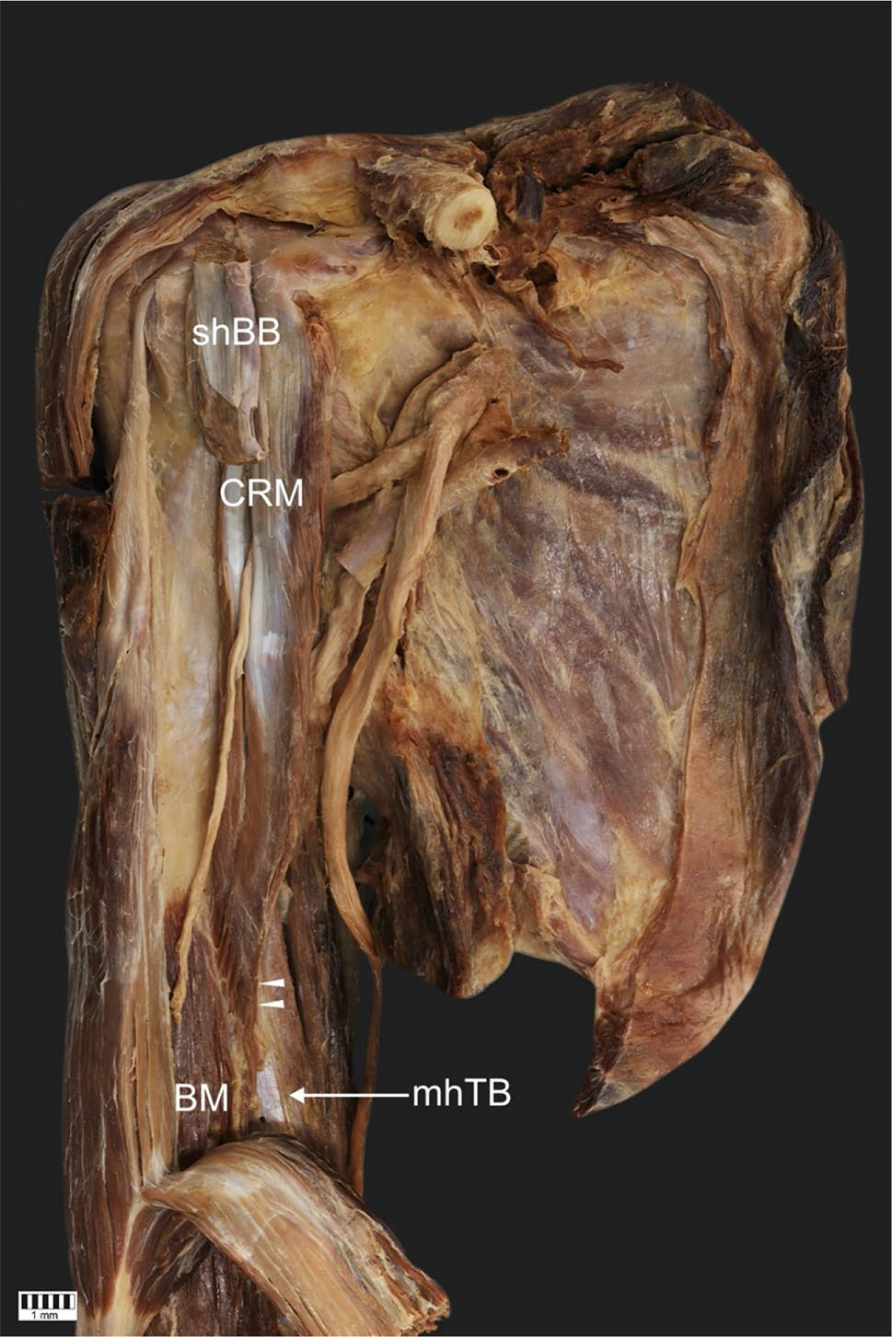
Type 1 of insertion of the coracobrachialis muscle. Right arm. *shBB* short head of the biceps brachii, *CRM* coracobrachialis muscle, *BM* brachialis muscle, *mhTB* medial head of the triceps brachii, *CP* coracoid process, *C* clavicle, white arrowheads show the insertion of the coracobrachialis muscle

**Fig. 6:**
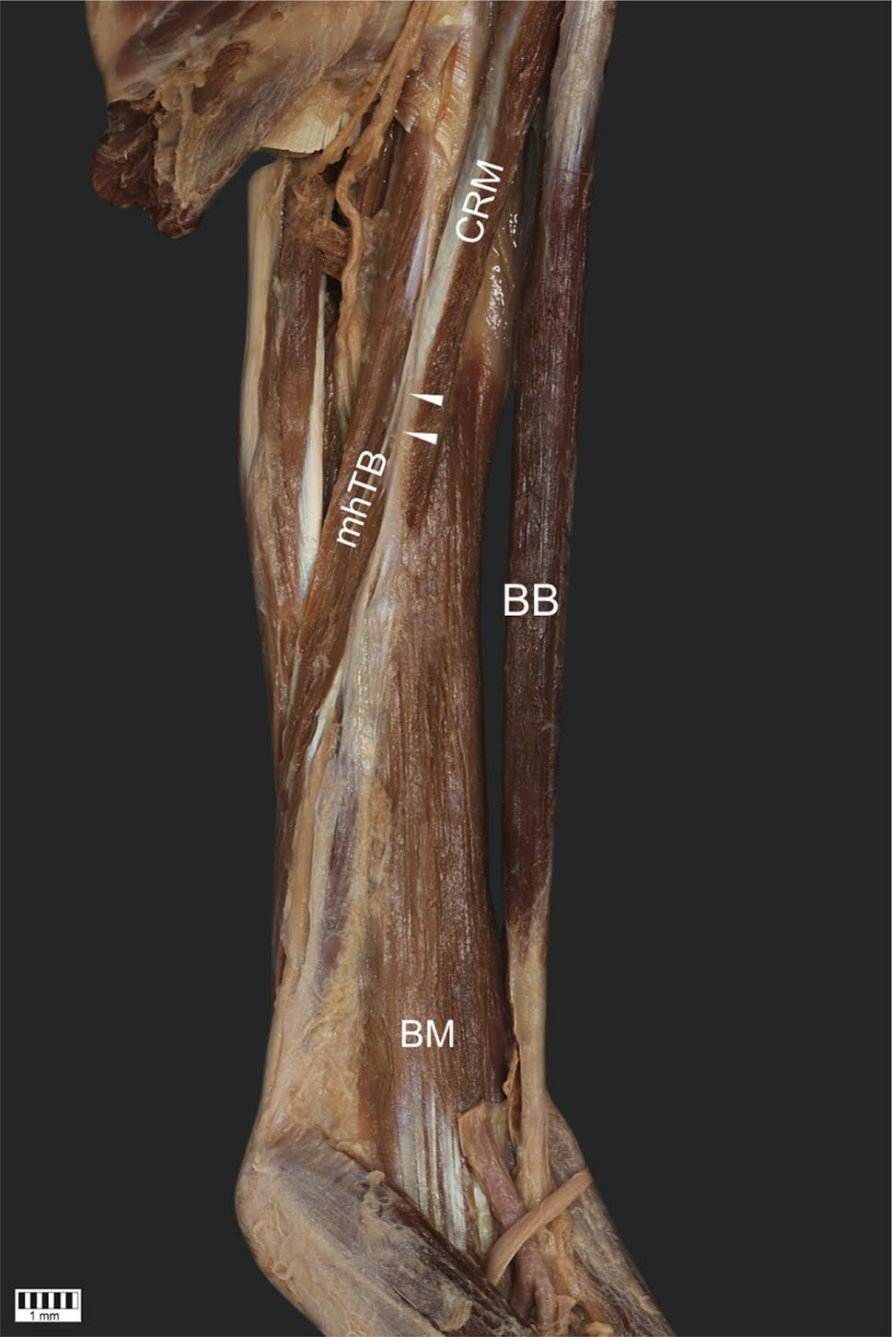
Type 2 of insertion of the coracobrachialis muscle. *mhTB* medial head of the triceps brachii, *CRM* coracobrachialis muscle, *BB* biceps brachii, *BM* brachialis muscle, *ME* medial epicondyle of the humerus, white arrowheads show fusion between coracobrachialis and medial head of the triceps brachii

The CRM was present in all 101 dissected limbs. The observed anatomical variations were grouped according to the following categories:

- Type of muscle origin
  - Type I (49.5%)—single belly originating from the coracoid process, medially and posteriorly to the tendon of the shBB (50 cases: 30 female and 20 male; 22 right and 28 left)—Figs. 1, 4a
  - Type II (42.6%)—double muscle belly originating from—Fig. 2.
    - Type IIa—one head originating from the coracoid process posteriorly to the tendon of the biceps brachii and a second head originating from the shBB (22 cases: 13 females and 9 males; 11 right and 11 left)—Figs. 2a, 4b.
    - Type IIb—both heads originating from the coracoid process; however, the superficial head fuses with the insertion of the shBB, while the deep head is directly originating (21 cases: 17 female and four male; 12 right and 9 left)—Figs. 2b, 4c.
  - Type III (7.9%)—three heads, two originating from the coracoid process (superficial and deep), whereas the third originates from the shBB (eight cases: eight males; four right and four left)—Figs. 3, 4d.
- Type of insertion
  - Type 1—single, classical insertion on the distal 1/3 of the humerus (61 cases: 32 female and 29 male; 29 right and 32 left)—Fig. 5.
  - Type 2—double insertion: one on the distal 1/3 of the humerus and another fusing with the medial head of the triceps brachii (40 cases: 28 females and 12 males; 20 right and 20 left)—Fig. 6.
- Relation to the MCN
  - Type I—it pierces the muscle belly (50 cases: 30 females and 20 males; 22 right and 28 left).
  - Type II—it passes between the heads of the coracobrachialis (51 cases: 30 females and 21 males; 27 right and 24 left).

A significant difference in origin type was observed between sexes (*p* = 0.0003) but not between body sides (*p* = 0.7861). However, no such differences were observed for insertion types (*p* = 0.1215 for sexes and *p* = 0.9495 for body sides) nor for the relationship with the MCN (*p* = 0.9344 for sexes and *p* = 0.4840 for body sides). Interestingly, only in one case of a double-headed CRM was the MCN found to pierce the muscle instead of passing between the two heads.

Morphometric parameters are presented according to sex and body side in Table 1, and according to insertion type and relationship to MCN in Table 2. In addition, they are presented with regard to origin type in Table 3.

**Table 1:** Morphometric parameters according to sex and bodyside

Parameter	Head	Sex		*P* value	Body side		*P* value
		Female	Male		Right	Left	
Muscle belly length	1	103.92 (18.32)	113.10 (19.27)	0.0211	107.84 (19.34)	107.46 (19.17)	0.7832
	2	87.85 (25.69)	81.94 (33.31)	0.4554	86.61 (27.41)	84.08 (31.04)	0.6989
	3		100.83 (12.65)	–	100.83 (12.61)	100.83 (14.64)	0.8852
Origin width	1	7.15 (2.24)	7.80 (1.72)	0.0424	7.45 (2.17)	7.38 (1.98)	0.6885
	2	9.81 (3.87)	11.15 (5.43)	0.7312	10.27 (4.74)	10.53 (4.54)	0.9718
	3		14.77 (3.69)	–	14.33 (3.92)	15.21 (3.98)	0.4705
Origin thickness	1	2.46 (0.70)	2.57 (0.66)	0.3330	2.50 (0.74)	2.51 (0.64)	0.9702
	2	1.76 (0.71)	1.87 (0.53)	0.2135	1.87 (0.68)	1.72 (0.59)	0.5169
	3		1.77 (1.41)	–	1.63 (1.20)	1.91 (1.77)	0.8852
Coracobrachialis tendon length	–	40.14 (13.08)	41.02 (14.70)	0.8900	42.02 (13.88)	39.06 (13.51)	0.2565
Muscle width in junction	–	5.00 (1.51)	5.70 (1.54)	0.0264	5.39 (1.50)	5.19 (1.61)	0.5319
Muscle thickness in junction	–	2.23 (0.58)	2.36 (1.04)	0.8600	2.32 (0.83)	2.26 (0.77)	0.4148
Distance between musculocutaneous nerve branching and piercing/passing through the muscle	–	69.36 (22.68)	74.23 (24.90)	0.3523	71.74 (23.74)	70.96 (23.71)	0.9107
Musculocutaneous nerve diameter	Before muscle	3.14 (1.08)	3.22 (0.86)	0.3742	3.25 (1.09)	3.10 (0.90)	0.5055
	After muscle	2.81 (1.03)	2.92 (0.82)	0.2822	2.96 (1.10)	2.75 (0.78)	0.4246

*p*-values lower than 0.0033 are significant, according to Bonferroni’s correction

**Table 2:** Morphometric parameters according to type of insertion and relationship with MCN

Parameter	Head	Type of insertion		*P* value	Relation to musculocutaneous nerve		*P* value
		Type 1	Type 2		Type 1	Type 2	
Muscle belly length	1	104.16 (19.29)	112.96 (17.91)	0.0327	104.24 (18.69)	110.98 (19.21)	0.0275
	2	70.43 (21.91)	101.01 (27.27)	0.0004	54.21 (0.00)	86.04 (28.85)	1.0000
	3	95.35 (8.94)	117.26 (0.89)	0.0668		100.83 (12.65)	–
Origin width	1	7.02 1.53 ()	8.01 (2.58)	0.0268	7.74 (2.33)	7.09 (1.72)	0.2987
	2	8.34 (2.93)	12.09 (5.07)	0.0017	11.21 (0.00)	10.37 (4.65)	–
	3	15.53 (4.02)	12.48 (0.71)	0.2433		14.77 (3.69)	–
Origin thickness	1	2.32 (0.60)	2.77 (0.73)	0.0020	2.48 (0.61)	2.53 (0.76)	0.4150
	2	1.91 (0.70)	1.71 (0.58)	0.1539	3.32 (0.00)	1.77 (0.60)	–
	3	1.04 (0.28)	3.96 (0.82)	0.0668		1.77 (1.41)	–
Coracobrachialis tendon length	–	43.01 (14.51)	36.67 (11.52)	0.0430	39.57 (14.84)	41.40 (12.56)	0.2801
Muscle width in junction	–	5.32 (1.50)	5.23 (1.65)	0.7337	5.26 (1.68)	5.31 (1.44)	0.8016
Muscle thickness in junction	–	2.27 (0.86)	2.32 (0.70)	0.5366	2.34 (0.84)	2.23 (0.75)	0.6152
Distance between musculocutaneous nerve branching and piercing/passing through the muscle	–	77.75 (24.20)	61.56 (19.12)	0.0012	71.00 (26.89)	71.67 (20.15)	0.6010
Musculocutaneous nerve diameter	Before muscle	3.01 (0.79)	3.42 (1.21)	0.0319	3.09 (0.89)	3.25 (1.09)	0.4016
	After muscle	2.78 (0.77)	2.96 (1.18)	0.4246	2.77 (0.88)	2.93 (1.02)	0.3131

*p*-values lower than 0.0033 are significant, according to Bonferroni’s correction

**Table 3:** Morphometric parameters according to origin type and insertion type

Parameter	Head	Type of origin				*P* value
		Type 1	Type 2		Type 3	
			a	b		
Muscle belly length	1	103.76 (19.16)	109.74 (23.52)	108.96 (14.39)	122.70 (6.28)	0.0281
	2	–	75.63 (31.14)	102.67 (16.57)	67.05 (26.32)	0.0020
	3	–	–	–	100.83 (12.65)	–
Origin width	1	7.80 (2.33)	6.97 (1.80)	6.70 (1.52)	8.09 (1.66)	0.1908
	2	–	11.24 (6.25)	11.02 (3.55)	7.14 (0.80)	0.0330
	3	–	–	–	14.77 (3.69)	–
Origin thickness	1	2.48 (0.61)	2.41 (0.76)	2.75 (0.78)	2.24 (0.61)	0.3363
	2	–	1.92 (0.83)	1.71 (0.54)	1.84 (0.48)	0.6988
	3	–	–	–	1.77 (1.41)	–
Coracobrachialis tendon length	–	39.50 (14.74)	46.00 (11.67)	40.44 (12.29)	31.72 (11.40)	0.0308
Muscle width in junction	–	5.31 (1.69)	5.44 (1.38)	5.32 (1.54)	4.64 (1.28)	0.6000
Muscle thickness in junction	–	2.33 (0.84)	2.03 (0.57)	2.52 (0.88)	2.13 (0.75)	0.4044
Distance between musculocutaneous nerve branch and passage through the muscle	–	71.60 (27.10)	72.00 (23.53)	66.73 (17.60)	79.93 (11.21)	0.4841
Musculocutaneous nerve diameter	Before muscle	3.09 (0.89)	2.92 (0.63)	3.59 (1.42)	3.35 (0.92)	0.1981
	After muscle	2.76 (0.88)	2.74 (0.62)	3.17 (1.41)	2.90 (0.48)	0.5463

*p*-values lower than 0.003 are significant according to Bonferroni’s correction

## Discussion

The key value of the present work is that it presents a new systematic classification of CRM origin and insertion based on anatomical dissection. In addition, it assesses the relationship between individual types of CRM and MCN.

To understand the occurrence of CRM variations, it is necessary to review their embryological development. Embryologically, the biceps brachii, CRM, and brachialis muscle are believed to arise from a common premuscular mass. The origins of the two heads of the biceps brachii become separated as the scapula develops. The three muscles can be recognized in embryos 14–16 mm in length, and the tendon of the long head in embryos 14 mm in length. The distal end of the common muscle mass differentiates later than the proximal end [20, 21]. The presence of the CBL could be explained as a result of the premature termination of this regression process.

Little information exists about the morphological variability of the proximal attachment of the CRM [2, 10]. Most likely, the first morphological variability of CRM was described by Wood [22], who describes coracocapsularis originating from the coracoid process and inserting into the shoulder capsule [22]. El-Naggar reports that the CRM consists of two heads; a superficial (anterior) head and a deep (posterior) head [2]. The superficial head originates from the medial border of the tendon of the shBB, while the deep head originates from the coracoid process of the scapula and the adjoining part of the lateral border of the tendon of the shBB [2]. The deep layer of the coracobrachialis can originate from the insertion of the pectoralis major [23]. Interestingly, one case has been reported of a three-headed CRM, characterized by a single superficial head and a deep head split into two [2]. In contrast, Ilayperuma et al. [10] do not report any such morphological variations in the CRM proximal attachment: they describe three possible proximal attachments for a single belly relative to the tendon of the biceps brachii[10], these being lateral to the tendon, medial to the tendon and deep to the origin of the tendon of the biceps brachii. Cases of accessory CRM have also been observed, which typically originates from the posterior margin of the coracoid process and inserts into the tendinous part of latissimus dorsi (the coracobrachialis minor or secundus) [22, 24]. Olewnik et al. [25] found a really rare case of CRM, which was characterized by four heads. The first two heads of the CRM demonstrate a proximal attachment at “the accessory apex” of the coracoid process, the third head, together with the head of the shBB, was attached to the apex of the coracoid process and was characterized by a fusion with the shBB; the fourth head was located under the head of the shBB and displayed an attachment at the inferior surface of the coracoid process [25]. The fourth head in the distal part demonstrated a fusion to the brachialis muscle, with the distal attachment being at the middle of the medial surface and the border of the body of the humerus, together with the other three heads [25].

In very rare cases, a coracobrachialis longus muscle may be observed [12, 13, 16], with the CRM being absent [22, 26].

However, a new classification is needed for clinical, anatomical and didactic reasons. The present study proposes a new threefold CRM classification (Types I-III), with Type II being further divided into two subtypes (A-B). The proposed classification is based on the number of bellies: Type I, present in 49.5% of cases, is characterized by a single belly with the proximal attachment located on the coracoid process, medially and posteriorly to the tendon of the shBB. Type II (42.6%) is characterized by the occurrence of two bellies. This type was divided into two subtypes: A and B. In Type IIA, the first head originates from the coracoid process posterior to the tendon of the shBB and the second head originates from the shBB. In Type IIB, both heads originate from the coracoid process; however, the superficial head fuses with the insertion of the shBB, while the deep head is directly inserted. This Type IIB corresponds to the type described by El-Nagger [2]. Type III is characterized by a three-headed CRM (7.9%): two heads (superficial and deep) originate from the coracoid process, whereas the third arises from the shBB. It is worth noting that this type of CRM has not been previously described in other studies on the variability of this muscle [2, 10].

It may seem that this small, inconspicuous muscle may have a much greater clinical significance than previously thought. It has been speculated that CRM is functionally not important; however, some studies suggest that it may be one of the most effective flexors of the shoulder joint and that it resists anterior dislocation [27]. The shoulder is the most regularly dislocated joint in the body, with dislocation occurring anteriorly, posteriorly, inferiorly, or anterior-superiorly. Of these, anterior locations are the most common, occurring in 95–97% of cases [28–30]. Patients with prior shoulder dislocation are more prone to redislocation, which typically occurs due to the tissue not healing properly or losing tension. Proximal rupture of the CRM can also hasten anterior dislocation of the shoulder [31]. It remains unclear, however, whether a CRM with two bellies is more likely to predispose the bearer to anterior dislocation than one with three bellies. Furthermore, the origin of the CRM from the shBB strongly indicates that CRM acts as a muscle enhancer for the shBB (Type IIa, III). A new CRM classification is needed. This would be a good step towards potentially extending the classification to "rare cases" in the upper and lower limbs, as was the case with earlier classifications [32–39].

The distal attachment of the CRM was usually observed on the medial border of the diaphysis of the humerus between the attachments of the medial head of the triceps brachii and the brachialis muscle [2, 10]. This type of insertion (Type 1) was observed in 60.4% of all cases in the present study. Type 2 characterized by a double insertion on the distal 1/3 of the humerus and fusion with the medial head of triceps brachii was observed in 39.6% of cases. Interestingly, it was found that the simultaneous proximal attachment to the shBB co-occurred with a simultaneous distal attachment to the medial head of the triceps brachii. However, the function of a muscle that demonstrates attachments to antagonistic muscles remains unclear.

Can a CRM with two or three bellies be used as a source of material in plastic surgery? Type IIa demonstrated the longest tendon (46 mm mean length) and could possibly be used to reconstruct other tendons and ligaments.

MCN neuropathy is not as common as MN, ulnar or radial neuropathy. It has a similar course to CRM neuropathy and can pierce or pass deep to the CRM [2, 7, 10, 40, 41]. The CRM is thought to be the most common site of MCN entrapment, and additional heads can place pressure on the MCN [4–6, 11, 12, 14]. MCN entrapment within the CRM muscle results in weakness and atrophy of the biceps brachii and brachialis muscles and a loss of sensation in the lateral forearm. Active young individuals that frequently engage in shoulder and elbow flexion with the forearm in a pronated position are most susceptible [42]. It also often occurs following chronic overuse of the CRM and consequent hypertrophy. No loss of CRM function will be observed as the nerve compressed within the CRM has already given off its motor branch to the CRM.

The present study outlines two types of innervation in relation to the CRM. In Type 1 the MCN pierces the CRM and is strongly associated with Type I muscle morphology (100% of cases). In contrast, in Types II and III, the MCN passes between the CRM heads. Interestingly, only in one case of a double CRM head did the MCN pierce the muscle instead of passing between the heads. The frequency of the atypical course and relationship of the MCN to the CRM has been exhaustively described in the literature [2, 3, 7, 10, 40, 41, 43]. The MCN innervates the CRM in 0 to 22% of cases [2, 4, 7, 10, 40, 41]. Interestingly, while previous studies found the CRM to not be pierced by the MCN in less than 7% of cases [8, 26, 44], the present study found it to be the case in 51%. It is possible that previous studies do not reveal the potential types of CRM or omit the deep layer, or that these differences result only from recently described population differences.

The present study has some limitations. One is the heterogeneous nature of the classification, which depends on several morphological details such as type of insertion or origin. In addition, it is only an anatomical study, and so a spectrum of variation could be presented; further studies should examine the potential value of ultrasound or MRI for this purpose. Nonetheless, this study helps raise awareness of what to look for, and where to find it, and offers a uniform classification and terminology, which can be used as a foundation for communication with surgeons.

Nevertheless, the proposed classification has four key assets. First, it recognizes the different possibilities of proximal attachment of the CRM. It also highlights the variety of distal attachments. It demonstrates the variable course of MCN relative to given types of CRM morphology (Type I-III). It also proposes a systematic classification of CRM morphological variability.

In addition, a thorough understanding of the CRM is needed for effective treatment and rehabilitation of anterior dislocation shoulder or MCN neuropathy, and hence our findings have offered new data for anatomy, physiotherapy, and orthopedic surgery. They can also be used in the future for the reconstruction of other tendons or ligaments, as well as support plastic surgery.

## Conclusion

The CRM is characterized by high morphological variability. The new classification proposes three types of proximal attachment (I–III) and two types of distal attachment. In addition, two types of MCN are distinguished. While Type I CRM is always pierced by the MCN, Types II and III are pierced in only one case. This inconspicuous muscle can be of great clinical importance, and our proposed classification may be of great value to surgeons operating in this area.

## Data Availability

Please contact authors for data requests (Łukasz Olewnik PhD—email address: lukasz.olewnik@umed.lodz.pl).
